# The use of artificial intelligence models to predict survival in patients with laryngeal squamous cell carcinoma

**DOI:** 10.1038/s41598-023-35627-1

**Published:** 2023-06-15

**Authors:** Nayeon Choi, Junghyun Kim, Heejun Yi, HeeJung Kim, Tae Hwan Kim, Myung Jin Chung, Migyeong Ji, Zero Kim, Young-Ik Son

**Affiliations:** 1 Department of Otorhinolaryngology, Head and Neck Surgery, Samsung Medical Center, Sungkyunkwan University School of Medicine, 81 Irwon-ro, Gangnam-gu, Seoul, 06351 Republic of Korea; 2 Medical AI Research Center, Research Institute for Future Medicine, Samsung Medical Center, Sungkyunkwan University School of Medicine, 81 Irwon-ro, Gangnam-gu, Seoul, 06351 Republic of Korea; 3 Department of Data Convergence and Future Medicine, Sungkyunkwan University School of Medicine, Seoul, Republic of Korea; 4 Department of Medical Device Management and Research, SAIHST, Samsung Medical Center, Sungkyunkwan University School of Medicine, 81 Irwon-ro, Gangnam-gu, Seoul, 06351 Republic of Korea

**Keywords:** Medical research, Oncology, Risk factors

## Abstract

Most recent survival prediction has been based on TNM staging, which does not provide individualized information. However, clinical factors including performance status, age, sex, and smoking might influence survival. Therefore, we used artificial intelligence (AI) to analyze various clinical factors to precisely predict the survival of patients with larynx squamous cell carcinoma (LSCC). We included patients with LSCC (N = 1026) who received definitive treatment from 2002 to 2020. Age, sex, smoking, alcohol consumption, Eastern Cooperative Oncology Group (ECOG) performance status, location of tumor, TNM stage, and treatment methods were analyzed using deep neural network (DNN) with multi-classification and regression, random survival forest (RSF), and Cox proportional hazards (COX-PH) model for prediction of overall survival. Each model was confirmed with five-fold cross validation, and performance was evaluated using linear slope, y-intercept, and C-index. The DNN with multi-classification model demonstrated the highest prediction power (1.000 ± 0.047, 0.126 ± 0.762, and 0.859 ± 0.018 for slope, y-intercept, and C-index, respectively), and the prediction survival curve showed the strongest agreement with the validation survival curve, followed by DNN with regression (0.731 ± 0.048, 9.659 ± 0.964, and 0.893 ± 0.017, respectively). The DNN model produced with only T/N staging showed the poorest survival prediction. When predicting the survival of LSCC patients, various clinical factors should be considered. In the present study, DNN with multi-class was shown to be an appropriate method for survival prediction. AI analysis may predict survival more accurately and improve oncologic outcomes.

## Introduction

Treatment of larynx cancer has significantly advanced with the evolution of radiation therapy technique, chemotherapeutic agents, surgical skill, and instruments^1–3^. The multidisciplinary approach has improved functional and oncologic treatment outcomes for larynx cancer but has variable clinical courses based on tumor burden and location.

Several staging manuals and treatment guidelines have been used to aid in treatment decision and survival prediction. Recently, survival estimation and treatment have been generally based on the 8th edition of the American Joint Committee on Cancer (AJCC) TNM staging system^4^, which shows reasonable accuracy. However, the AJCC TNM staging system does not provide individualized information that could enhance accurate survival prediction and successful oncologic outcomes with reduced morbidity.

Survival prediction and treatment strategy are especially difficult in larynx cancer due to its heterogeneous histology; various subsites (including supraglottis, glottis, and subglottis); and performance status, and treatment methods. In addition, survival could be influenced by age, biologic and genomic features, and other comorbidities^5^.

Several prognostic calculators have been used for head and neck cancer^5–8^. In most studies, conventional regression statistical models have been widely used to estimate the linear relationships between clinical variables for survival estimation. However, many clinical factors associated with survival of larynx cancer do not have mathematical linearity, and accurate survival prediction is difficult when using regression models^9–11^.

Recently, artificial intelligence (AI) has been developed for prediction of survival in various cancers associated with linear and non-linear variables^12–16^. Due to the development of AI technology, interpretive quantitative analysis was performed in various machine learning systems. Cox proportional-hazards (COX-PH), random survival forest (RSF), and deep neural network (DNN) algorithms were used in this analysis. The COX-PH model is a statistical regression model commonly used in medical research to investigate the association between the survival time of patients and one or more predictor variables^17^. RSF models have been identified as alternative methods to the COX-PH model for analyzing time-to-event data^18^. The DNN algorithm is a further evolution of machine learning that emulates the synaptic structure of neurons in the brain and consists of input and output layers and one or more hidden layers between them. The DNN learns complex relationships between input variables that have nonlinear characteristics. The first input layer passes input data to the next layer with a full connection, where the node of each layer is a weighted linear combination of the output of the previous layer nodes. Then, the output of each node is transformed by a nonlinear function. This connection repeats until reaching the output layer.

The aims of this study were to develop a survival prediction model using COX-PH, RSF, DNN for patients with laryngeal squamous cell carcinoma (LSCC) treated in a single tertiary center. The clinical factors used for development of AI models for general application were easily accessible variables including age, sex, TNM staging, performance status, treatment methods, and recurrence pattern.

## Results

### Clinical and pathological characteristics of the patients who received definitive treatment for laryngeal squamous cell carcinoma

The patients with LSCC (n = 1020) were analyzed (Table 1). The mean age was 64.5 ± 9.7 years, and the subjects were predominantly male (n = 979, 96.0%). Current smokers (n = 25, 24.5%) were most common, followed by ex-smokers (n = 209, 20.5%) and non-smokers (n = 88, 8.6%). Alcohol consumption included none (n = 104, 10.2%), ex-drinker (n = 244, 23.9%), and current drinker (n = 168, 16.5%). Smoking and alcohol consumption data of 473 (46.4%) and 515 (49.4%) patients, respectively, could not be obtained.

**Table 1:** Clinical variables of patients with laryngeal squamous cell carcinoma (n = 1020).

Clinical variables	
Age (years), mean ± SD	64.5 ± 9.7
Sex: M, F, n (%)	979, 41 (96.0, 4.0)
Smoking status: non-smoker, ex-smoker, current smoker, unknown, n (%)	88, 209, 250, 473 (8.6, 20.5, 24.5, 46.4)
Alcohol consumption: non-drinker, ex-drinker, current drinker, unknown, n (%)	104, 244, 168, 515 (10.2, 23.9, 16.5, 49.4)
ECOG performance status: 0, 1, 2, 3, 4, unknown, n (%)	468, 283, 63, 15, 7, 184 (45.9, 27.7, 6.2, 1.5, 0.7, 16.1)
Tumor location, n (%)	
Supraglottis	231 (22.6)
Glottis	767 (75.2)
Subglottis	22 (0.22)
Tumor staging, n (%)	
T1, 2, 3, 4	592, 199, 136, 93 (58.0, 19.5, 13.3, 9.1)
N0, 1, 2a, 2b, 2c, 3a, 3b	837, 41, 10, 70, 43, 3, 16 (82.1, 4.0, 1.0, 6.9, 4.2, 0.3, 1.6)
Stage I, II, III, IV	578, 144, 97, 201 (56.7, 14.1, 9.5, 19.7)

*ECOG* Eastern Cooperative Oncology Group, *SD* standard deviation.

The most common tumor location was the glottis (n = 767, 75.2%) followed by supraglottis (n = 231, 22.6%) and subglottis (n = 22, 0.22%). The number and percentage of T1, T2, T3, and T4 stages were 592, 199, 136, and 93 and 58.0%, 19.5%, 13.3%, and 9.1%, respectively. Those of N0, N1, N2a, N2c, N3a, and N3b stages were 837, 41, 10, 70, 43, 3, and 16 and 82.1%, 4.0%, 1.0%, 6.9%, 4.2%, 0.3%, and 1.6%, respectively.

### Treatment methods and oncologic outcomes of patients with laryngeal squamous cell carcinoma

Surgery only was the most common treatment method (n = 373, 36.6%) because early glottic cancer (T1/T2N0) was predominant in our cohort (Table 2). Surgery with adjuvant radiation was administered to 201 (19.7%) patients, surgery with adjuvant chemoradiation to 38 (3.7%) patients, only radiation to 294 (28.8%) patients, and concurrent chemoradiation to 114 (11.2%) patients.

**Table 2:** Treatment methods and oncologic outcomes of patients who received definitive treatment for laryngeal squamous cell carcinoma (n = 1020).

Clinical variables	
Treatment methods, n (%)	
Surgery	373 (36.6)
Surgery + adjuvant RT	201 (19.7)
Surgery + adjuvant CCRT	38 (3.7)
RT	294 (28.8)
CCRT	114 (11.2)
Recurrence, n (%)	200 (19.6)
Local recurrence, n (%)	142 (13.9)
Regional recurrence, n (%)	53 (5.2)
Distant metastasis, n (%)	36 (3.5)
Death, n (%)	154 (15.1)
Time to recurrence, months, mean ± SD (range)	15.6 ± 16.0 (3–59)
Time to death, months, mean ± SD (range)	40.4 ± 19.3 (3–60)

*RT* radiation therapy, *CCRT* concurrent chemoradiation therapy, *SD* standard deviation.

Recurrence was observed in 200 (19.6%) patients, consisting of local (n = 142, 13.9%), regional recurrence (n = 53, 5.2%), and distant metastasis (n = 46, 3.5%). Death occurred in 154 (15.1%) patients. Mean time to recurrence was 15.6 ± 16.0 months (minimum–maximum, 3–59 months), and mean time to death was 40.4 ± 19.3 months (minimum–maximum, 3–60 months).

**Figure 1:**
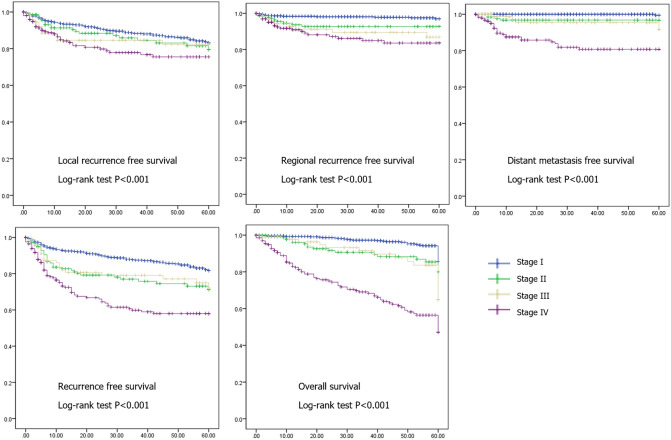
Analysis of recurrence-free survival and overall survival using Kaplan–Meier analysis with log-rank test in patients with larynx squamous cell carcinoma based on tumor stage (n = 1020).

In analysis based on tumor stage, local recurrence-free survival, regional recurrence-free survival, distant metastasis-free survival, and overall survival were significantly different (p < 0.001) by tumor stage (Fig. 1). Fiver-year local recurrence free survival rates were 82.9%, 79.1%, 81.1%. and 74.4%, and regional recurrence free survival rates were 96.5%, 90.0%, 88.1% and 84.1% in stage I, II, III and IV, respectively. Five-year distant metastasis free survival rates were 99.1%, 91.3%, 90.9%, and 81.3%, and overall recurrence free survival rates were 81.8%, 66.3%, 70.3%, and 59.3% in stage I, II, III and IV, respectively. Five-year overall survival rates were 84.6%, 72.4%, 54.2%, and 53.9% in stage I, II, III and IV, respectively.

### Performance analysis using artificial intelligence algorithms

**Figure 2:**
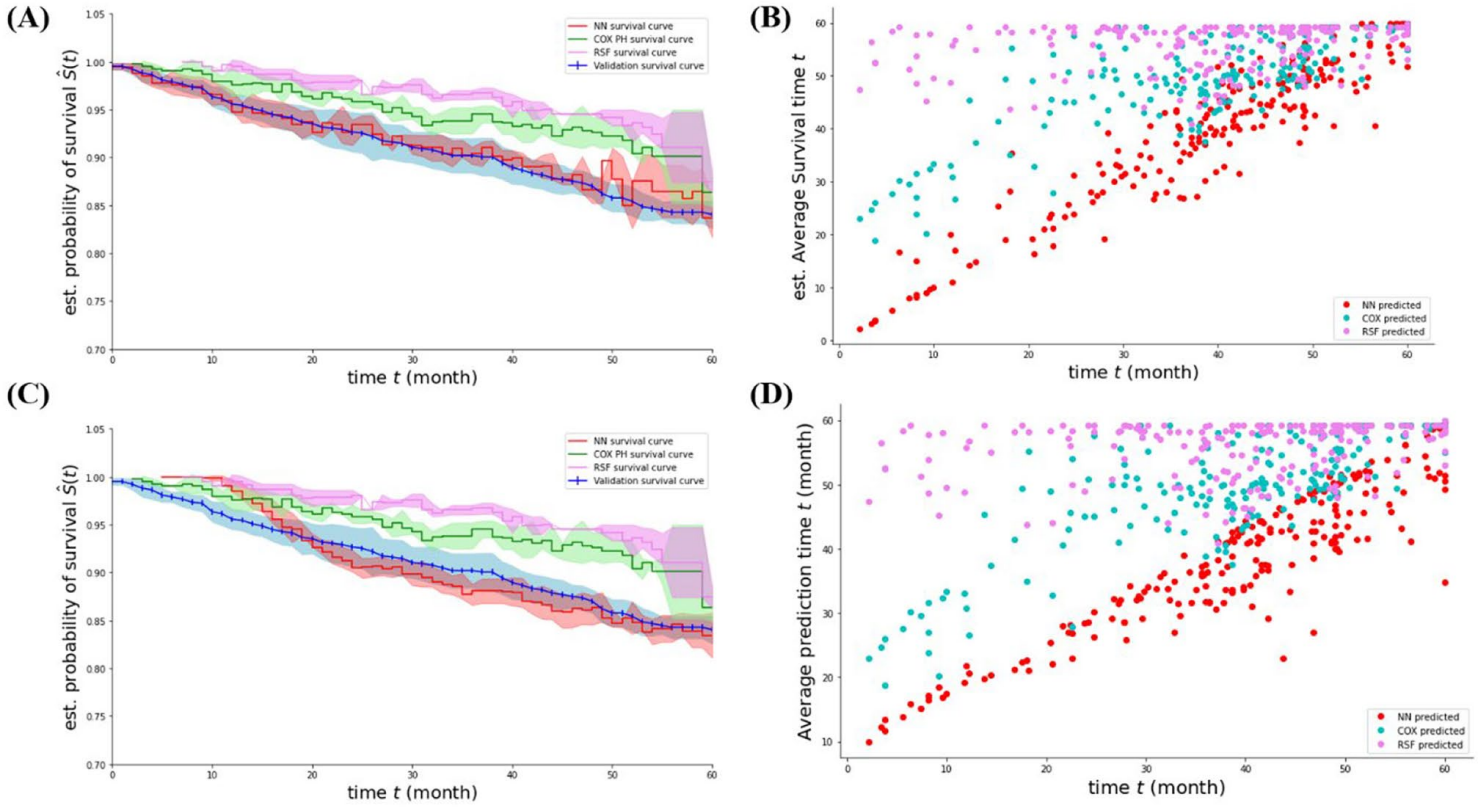
Estimated survival curves and scatter plots of artificial intelligence models of the patients with laryngeal squamous cell carcinoma. (**A**,**B**) Comparison between validation, Cox proportional hazard model, random survival forest, and deep neural network with multi-classification methods. (**C**,**D**) Comparison between validation, Cox proportional hazard model, random survival forest, and deep neural network with regression methods.

The survival period and mortality results using the four AI prediction algorithms are summarized in Table 3 and Fig. 2. The average concordance indices of survival period predictions were 0.747 ± 0.009 from COX-PH, 0.596 ± 0.015 from RSF, 0.893 ± 0.017 from DNN regression, and 0.859 ± 0.018 from DNN multi-classification. The linearity test was applied because the DNN regression results and multi-classification values were within the standard deviation, and concordance index cannot demonstrate an increase in the survival period or exact prediction of the survival period^19^. The average slope and y-axis were 0.731 ± 0.048 and 9.659 ± 0.964 for DNN regression and 1.000 ± 0.047 and 0.126 ± 0.762 for DNN multi-classification, respectively. Significant discrimination was found between the DNN regression results and DNN multi-classification using the linearity test. The micro-average AUC of survival period prediction was 0.937 ± 0.011 from the DNN multi-classification for 60 months. The average AUCs for mortality were 0.682 ± 0.055 with DNN regression and 0.841 ± 0.020 with DNN multi-classification.

**Table 3:** Predictive performance of artificial intelligence models for overall survival of patients who received definitive treatment for laryngeal squamous cell carcinoma (n = 1020).

Prediction model	Linear slope	Linearity-y-intercept	C-index
Deep neural network with regression	0.731 ± 0.048	9.659 ± 0.964	0.893 ± 0.017
Cox proportional hazard model	0.619 ± 0.058	24.483 ± 2.407	0.747 ± 0.009
Random survival forest	0.079 ± 0.057	53.250 ± 2.349	0.596 ± 0.015
Deep neural network with multi-classification	1.000 ± 0.047	0.126 ± 0.762	0.859 ± 0.018
Deep neural network with only T/N staging	Not available	Not available	0.504 ± 0.007

In addition, the average concordance index of the survival period predictions for T/N only with DNN multi-classification was 0.504 ± 0.007. The slope and y-axis for T/N cannot be calculated using DNN multi-classification because of poor predictive performance.

We analyzed the patients with glottic cancer separately, which is the largest proportion in our data (Supplementary Fig. S1). However, the prediction performance of the glottic cancer patients only decreased compared to it of the larynx cancer patients. In the analysis of the patients with glottic cancer, the average concordance indices of survival period predictions were 0.694 ± 0.019 from COX-PH, 0.539 ± 0.007 from RSF, 0.875 ± 0.021 from DNN regression, and 0.865 ± 0.021 from DNN multi-classification. The average slope and y-axis were 0.609 ± 0.038 and 15.665 ± 0.537 for DNN regression, 0.996 ± 0.047 and 0.572 ± 1.121 for DNN multi-classification, 0.372 ± 0.047 and 37.810 ± 2.487 for COX-PH, − 0.004 ± 0.016 and 58.685 ± 0.451 for RSF, respectively. This decline in predictive performance appears to be due to a significant decrease in the number of those with glottic cancer only (n = 767) compared to the patients with whole larynx cancer (n = 1020).

## Discussion

LSCC is one of the most prevalent head and neck cancers. The 5-year overall survival rate was approximately 60% for laryngeal cancer^20^. The 8th edition of AJCC TNM staging has recently provided key information for predicting prognosis and a basis for treatment decision^4^. However, other survival-related clinical factors, including clinicopathological and genomic data, were not considered in the current staging manual, resulting in a lower than expected prediction power. Therefore, accurate survival prediction with consideration of clinical factors as well as tumor staging is important to optimize treatment and improve oncologic outcomes. In this study, we developed statistical survival prediction models for LSCC using various clinical factors generally available in the clinical field. We demonstrated poor accuracy of survival prediction with tumor staging (C-index, 0.504 ± 0.007); however, survival prediction using tumor staging and various clinical factors (age, sex, treatment methods, recurrence, smoking, alcohol consumption, tumor location, and performance status) using a DNN with multi-classification showed significantly better performance (C-index, 0.859 ± 0.018).

Tumor staging, age, and performance status are well known survival predictors in cancer patients^4,21–25^. In addition, smoking and alcohol consumption were suggested as prognostic factors as well as major risk factors for development of larynx cancer^26–28^. Tumor location can also affect oncologic outcomes and treatment methods for patients with laryngeal cancer. In several studies, patients with supraglottic cancer showed poor survival compared with patients with glottic and subglottic cancer^28,29^.

The survival outcomes based on treatment method (surgical treatment and non-surgical organ preservation treatment based on radiation therapy) for larynx cancer are a controversial issue, and treatment methods should be analyzed for accurate prediction of survival. With development of organ preservation treatment, multiple treatment modalities have been applied for larynx cancer. Radiation-based therapy has been the major treatment option for early and advanced larynx cancer^30–32^. However, several conflicting results regarding the clinical outcomes of surgical treatment and non-surgical treatment were reported in previous studies^22,28,30–33^. In addition, the previous studies had several limitations because heterogeneous tumor staging manuals were used during the study period, and the patients had different clinicopathological characteristics including histologic types, subsites, tumor stage, and performance status.

Recurrence is an important predictor of disease-specific survival^28,34^. Patients with recurrent laryngeal cancer could have cancer and salvage/palliative treatment-related complications that can result in poor survival. Therefore, for accurate prediction of survival outcome, recurrence should be considered.

Several trials have been conducted for accurate survival prediction of larynx cancer considering various clinical factors and TNM staging. In most previous studies, the prediction models developed were based on Cox regression analysis^7,8,33^. In a recent study of population-based prediction models using Cox regression analysis, the survival model using multiple parameters (age, sex, T/N stage, tumor grade) achieved a C-index of 0.602, while a survival model using only T/N stage had a C-index of 0.547^33^. However, predictive performance in previous models has been poorer than expected. In our study, accuracy of survival prediction significantly increased when analyzed with multiple clinical factors compared with analysis only with T/N stage. In addition, AI technology was used to improve predictive performance of survival models in LSCC.

Conventional regression such as COX-PH, DNN, and RSF, which have been used in survival prediction studies, are not used for classifying data because they are sensitive to outliers. Determining the probability in linear regression models is difficult. The linear regression model cannot interpret the prediction as a probability because the prediction is simply interpolated between points.

Recently, several studies have applicated artificial intelligence for more accurate cancer survival prediction^35–37^. We also applicated the RSF, DNN and DNN with multi-classification. The advantage of DNN with multi-classification compared to DNN with regression is to extend estimators to approximate a series of target functions. The model is trained on a single predictor matrix to predict a series of responses, interpret the predicted values as a probability, and describe the bounds.

In this study, we developed more accurate survival prediction models for larynx cancer and compared it with conventional models. In addition, we used clinical factors that can be easily collected, and the models could be widely used for prediction of survival, surveillance after treatment, and treatment decision. However, our study also has several limitations. First, this study was designed as a single-center, retrospective study and requires external validation. To overcome this limitation, we performed five-fold cross-validation in multiple AI models. Next, treatment methods including surgery and radiation were developed during the study period, which could cause bias.

In our study, we introduced survival prediction models for LSCC using DNN with multi-class with easily accessible clinical variables. We expect that accurate survival prediction for LSCC and proper treatment decision could be possible through this model.

## Materials and methods

### Study population

We reviewed the medical records of patients who received definitive treatment for LSCC between 2002 and 2020 at our institution. The Institutional Review Board of Samsung Medical Center approved this retrospective study. The institutional Review Board of Samsung Medical Center waived the need for informed consent. All methods were performed in accordance with the relevant guidelines and regulations. During the study period, 1,626 patients received treatment for pathologically proven LSCC; and patients lost to follow-up, treated at other hospitals (n = 460), salvage or palliative cases (n = 76), initial M1 (n = 26), non-LSCC, and double primary cancer cases (n = 44) were excluded. Finally, we enrolled 1,020 patients who received definitive treatment for LSCC.

### Pretreatment work-up, treatment, and follow-up

The patients underwent laryngoscopic examination, neck enhanced computed tomography (CT), and positron emission tomography (PET)-CT for pre-treatment evaluation. The primary tumor was pathologically diagnosed based on biopsy under local or general anesthesia. Ultrasonography-guided fine needle aspiration biopsy was performed if cervical lymph node metastasis was suspected. Surgery was performed to eradicate any laryngeal tumor with negative resection margin. Adjuvant therapy after surgery was performed based on NCCN guidelines. Radiation therapy was performed mainly using intensity modulated radiation treatment based on institutional protocol^38^. Chemotherapy was mostly performed with two tri-weekly cycles of cisplatin (100 mg/m^2^)-based regimen.

For regular check-ups, patients visited the hospital at approximately 3–6 months intervals. At each visit, the patients received history taking, physical examination, endoscopic examination, and neck CT. Chest CT or PET-CT was performed at 12–18 months after treatment completion.

### Clinical factors for prediction of survival

We evaluated clinical variables of age, sex, smoking, alcohol consumption, Eastern Cooperative Oncology Group (ECOG) performance status, tumor subsites, and tumor stage. Smoking status and alcohol consumption were classified into current smoker/drinker (lifetime and within the last month), ex-smoker/drinker (lifetime but not within the last month), and never smoker/drinker. Performance status was determined based on ECOG performance status grade^39^.

Tumor subsites were classified into supraglottis, glottis, and subglottis. For analysis with a recent staging method, tumor stage was defined based on the AJCC (8th edition) TNM staging manual.

In addition, treatment methods and oncologic outcomes were collected. Treatment methods were categorized into surgery only, surgery with radiation therapy, surgery with concurrent chemoradiation therapy, radiation therapy, and concurrent chemoradiation therapy. Recurrence and death were investigated, and recurrence was divided into local, regional, and distant. Times to recurrence and to death from the start of treatment were calculated.

### Kaplan–Meier analysis with log-rank test for enrolled patients based on tumor stage

Recurrence-free survival (overall, local, regional recurrence, and distant metastasis) and overall survival were analyzed using Kaplan–Meier with log-rank test based on tumor stage. Statistical analysis was performed using SPSS version 25.0, and p-value < 0.05 was considered statistically significant.

### Artificial intelligence analyses

#### Encoding of clinical factors for artificial intelligence analysis

The one-hot encoding method was used to encode categorical variables because there was no ordinal relationship between the clinical factors. Using the encoding technique, categorical variables were transformed into binary variables for each category, only one of which was assigned a positive value to avoid misunderstanding of a categorical variable as an ordinal integer variable when performing AI analysis. After preprocessing, 40 encoded variables were used as input variables for AI algorithms.

#### Artificial intelligence algorithms

Data learning was performed using COX-PH, RSF, and DNN algorithms that consider the nonlinearity among variables and have the advantage of automatically learning data characteristics without having to set features directly. The DNN model was used for both regression and multi-classification for 60-month survival. Simultaneously, binary classification was performed for mortality prediction of each subject in the DNN model. The probability value of each class was inferred by applying “SoftMax” to the output layer of the DNN binary classification and DNN multi-classification models. We applied “mse” to the output layer of the DNN regression model.

We used 16 features of COX-PH, RSF, and DNN. Both dependent and independent features were used for learning because linearity was not assumed and the multi-collinearity effect on prediction of features was included in the neural network. We optimized neural network hyperparameters of number of layers, number of nodes, batch size, and learning rates by performing a grid search. The AI-based models for survival period and mortality prediction were developed using Python software as well as TensorFlow, Keras, and Scikit-learn libraries.

#### Evaluation of artificial intelligence models

A stratified five-fold cross-validation test was utilized to evaluate the performance of the machine learning models trained with four of the partitions and tested with the remaining partition. The validation test was performed five times for each of the five partitions, and the entire cross-validation process was repeated five times with a random split of the dataset^40^. A concordance index was used to evaluate the performance of AI models based on survival period prediction. The concordance index is a goodness-of-fit measure for models that produce risk scores and are commonly used to evaluate risk models in survival period analysis in which data may be censored. The DNN model has an advantage that it contains a module to obtain the concordance index without calculating risk score^41^. In addition, the linearity test, a technique from statistics, physics, and medical laboratory tests, was applied. Linearity is the ability to provide training results that are directly proportional to the concentration of the measurement (quantity to be measured). Linearity test is defined as y = ax + b, where x and y denote measurement and prediction, respectively, and a and b denote slope and y-axis. Linearity is a measure of training or fit bias between expectation and measurement. The ideal case of linearity is parameters a = 1 and b = 0^42^. An AUC was used to evaluate DNN model performance of mortality prediction and DNN multi-classification model.

## Supplementary Information

Supplementary Information 1.

Supplementary Information 2.

## Data Availability

The data supporting the findings of this study are available from the corresponding author upon reasonable request.
