# Supplementary material for: The use of artificial intelligence models to predict survival in patients with laryngeal squamous cell carcinoma
Source: Sci Rep. 2023 Jun 15;13:9734. doi: 10.1038/s41598-023-35627-1 (PMC10272182; doi:10.1038/s41598-023-35627-1)
Supplement: Supplementary file 1 — Supplementary Information 1. [file 41598_2023_35627_MOESM1_ESM.docx]

Supplementary Figure 1. Estimated survival curves of artificial intelligence models of the patients with glottic cancer only (n = 767)
