# Supplementary figures and images for: The use of artificial intelligence models to predict survival in patients with laryngeal squamous cell carcinoma
Source: Sci Rep. 2023 Jun 15;13:9734. doi: 10.1038/s41598-023-35627-1 (PMC10272182; doi:10.1038/s41598-023-35627-1)

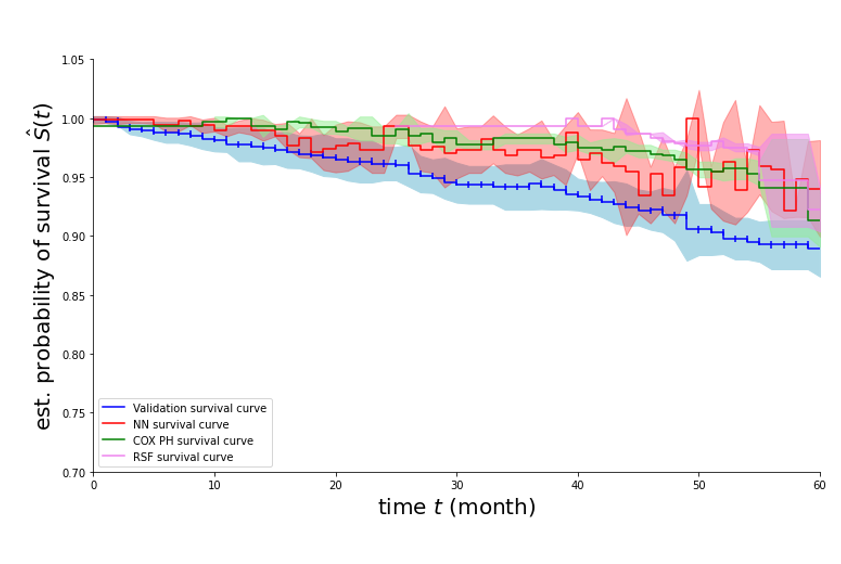

Supplement: Supplementary file 2 — Supplementary Information 2. [file 41598_2023_35627_MOESM2_ESM.png]
